# Establishment and validation of porcine embryo genomic selection system through SCNT technique

**DOI:** 10.1186/s40104-026-01514-8

**Published:** 2026-10-08

**Authors:** Songxian Zhou, Jianhua Wang, Xueyi Ding, Zhiyuan Lin, Yan Shang, Wenting Song, Hongfei Duan, Tingting Wang, Kai Zhou, Jingjing Zhang, Xihong Tan, Fang Fang, Lilin Yin, Yiliang Miao, Guojian Ma, Xin Liu

**Affiliations:** 1 Institute of Stem Cell and Regenerative Biology, College of Animal Science and Veterinary Medicine, Huazhong Agricultural University, Wuhan, 430070 China; 2 Key Laboratory of Agricultural Animal Genetics, Breeding and Reproduction (Huazhong Agricultural University), Ministry of Education, Wuhan, 430070 China; 3 Breeding Department, Wuhan COFCO Meat Co., Ltd., Wuhan, Hubei 430074 China; 4 Hubei Hongshan Laboratory, Wuhan, 430070 China

**Keywords:** Embryo genomic selection, GEBV prediction, Pig, SCNT

## Abstract

**Background:**

Embryo genomic selection (EGS) refers to the technique of performing genotyping and genomic prediction at the preimplantation stage. When combined with in vitro embryo production method, EGS enhances selection intensity and offers the potential to prevent the birth of offspring carrying genetic defects or an undesired sex. Given that the somatic cell nuclear transfer (SCNT) technique can copy the genetic information from donor pigs into early embryos, this study aims to establish and validate a complete porcine EGS workflow by integrating SCNT with genomic selection methodology.

**Results:**

First, porcine SCNT embryos were constructed using donor cells collected from candidate pigs with known genotypes and phenotypes. Cell biopsy performed in SCNT embryos revealed that collecting approximately 9.5 trophectoderm (TE) cells from blastocysts had the least impact on subsequent development. Next, a whole-genome multiple displacement amplification protocol was optimized for TE biopsy cells to conduct 80 K single nucleotide polymorphism (SNP) array analysis. The SNP call rates ranged from 94.44% to 99.77%, with genotype concordance between SCNT embryos and their donor pigs mostly exceeding 95%. Finally, genomic estimated breeding values (GEBVs) for three production traits were calculated using the GBLUP and Bayesian methods, and strong positive correlations (*r* > 0.90) of GEBVs were observed for each embryo and its donor.

**Conclusions:**

This study establishes and validates a complete EGS workflow for pigs, including embryo biopsy, whole-genome amplification, SNP genotyping, and GEBV prediction. The workflow provides a technical foundation for applying EGS in pig breeding and has the potential to accelerate genetic improvement through early embryo selection. Additionally, the results demonstrate that SCNT can be used to replicate elite genetic resources for the pig farming industry.

**Supplementary Information:**

The online version contains supplementary material available at https://doi.org/10.1186/s40104-026-01514-8.

## Introduction

Livestock breeding is the fundamental approach to enhancing genetic progress with good performance, which advanced the livestock agriculture by increasing the efficiency of meat and milk production. To date, breeding strategies have been updated by several technological generations. Early selection depended entirely on phenotypic observations, until the development of best linear unbiased prediction (BLUP), which integrated phenotypic data with pedigree information [1]. Subsequent advances included marker-assisted selection and the development of genomic selection (GS) introduced by Meuwissen in 2001 [2]. GS utilizes high-density single nucleotide polymorphism (SNP) markers distributed across the entire genome to construct statistical models that link genotypes to phenotypes, thereby enabling the prediction of genomic estimated breeding values (GEBVs) for individual animals. Previous studies have used SNP chips of varying densities to genotype large dairy cattle populations and to evaluate the accuracy of genotype prediction [3]. In addition, high-density SNP chips have been applied to assess genomic diversity in European local pig breeds and wild boars, providing insights into genetic differences and informing conservation strategies [4, 5]. However, conventional GS still relies on post-natal sampling and genotyping. This creates a time lag that hinders further reduction of the generation interval, ultimately constraining the efficiency of genetic progress [6]. Moreover, it is difficult to preemptively identify individuals with chromosomal abnormalities, potential genetic risks, or low breeding values. This limitation leads to increased costs associated with inefficient reproduction.

To address these limitations, embryonic genomic selection (EGS) has been proposed. This approach involves testing and evaluating the genomes from preimplantation embryos. Embryos with normal chromosomal ploidy, high breeding values for target traits, and an absence of deleterious mutations are then selected for embryo transfer. Consequently, EGS reduces the incidence of stillbirths and congenital abnormalities while preventing the birth of low-performing individuals with undesired sex. When combined with reproductive technologies, such as superovulation and in vitro embryo production, EGS enables the genotyping of a large pool of candidate embryos. Expanding this candidate pool not only increases selection intensity but also significantly enhances genetic diversity. Above all, EGS can accelerate genetic improvement in livestock and substantially improve the efficiency of elite breeding programs.

The conceptual foundation of EGS can be traced back to the applications of in situ hybridization for Y chromosome identification in biopsied bovine embryos [7]. Recently, integrating SNP chip genotyping with embryo biopsy has established a comprehensive EGS framework in ruminant breeding [8–14]. The typical procedure involves performing micromanipulation-based cutting or biopsy on the embryo, followed by whole-genome amplification (WGA). The amplified DNAs are then genotyped using SNP chips to enable accurate estimation of genomic breeding values. For instance, Silvestri et al. [15] utilized genome-wide SNP analysis to reveal chromosomal abnormalities in 5.0% to 14.1% of bovine embryos. Euploid embryos achieved a pregnancy rate of 59.6%, whereas aneuploid embryos exhibited a markedly lower rate of 5.8%, highlighting the critical role of genetic screening in early embryos. In 2025, EGS was successfully established in sheep [13]. Trophectoderm (TE) biopsy combined with a 50 K SNP chip achieved a detection rate of 93.71%, and this could be increased to approximately 98% when combined with whole-genome sequencing and imputation. Furthermore, GEBVs for several traits showed strong concordance (*r* = 0.91–0.98) between embryonic and the postnatal lambs, providing robust technical support for EGS in sheep breeding.

By contrast, no studies have reported the establishment of EGS in pigs. The application of EGS in pig breeding has been limited by several challenges: the absence of stable biopsy strategies, as well as reliable WGA protocols for porcine embryos, and no validation linking embryo and its offspring for genetic evaluation. To address these, the present study employed the somatic cell nuclear transfer (SCNT) technique to generate cloned embryos that are genetically identical to donor pigs. These cloned embryos served as a robust model to test embryo sampling strategies, WGA methods, SNP chip-based genotyping and breeding value estimation. Our results demonstrated that SNP genotypes obtained from cloned embryos exhibited a concordance rate of 95.76% with those from donor pigs. Furthermore, GEBVs for three production traits also showed positive correlations between embryos and donors. Together, these findings provide and validate a complete EGS workflow for pig breeding.

## Materials and methods

### Fibroblast and oocyte collection

Duroc and Yorkshire boars (120–130 days after birth) of the candidate group were provided for ear tissue collection by the Wuhan COFCO Meat Co., Ltd. 1 mm^2^ ear tissues were cut and transported to the laboratory, washed with phosphate buffer saline (PBS) and 75% ethanol, with epidermis and cartilage removed, and then minced and digested overnight with 0.25% trypsin at 4 °C. After centrifugation, porcine fibroblasts were cultured in DMEM medium containing 10% fetal bovine serum (FBS) at 37 °C in a 5% CO_2_ incubator (Thermo Scientific, Forma II) and were 1:2–1:3 split cultured at 70%–90% confluence. For cell cryopreservation, fibroblasts were digested and resuspended in freezing medium (90% FBS + 10% DMSO), pre-cooled at −80 °C, and stored in liquid nitrogen. For cell recovery, fibroblasts were rapidly thawed in a 37 °C water bath, then centrifuged and resuspended in DMEM medium containing 10% FBS, and cultured at 37 °C in a 5% CO_2_ incubator.

Ovaries were collected from three-way crossbred pigs [Duroc × (Landrace × Yorkshire), DLY] at a local slaughterhouse, placed in pre-warmed 37 °C saline and transported to the laboratory within 2 h. Follicular fluid from 2–6 mm follicles was aspirated for picking cumulus–oocyte complexes, and these complexes were cultured in in vitro maturation medium [16] at 38.5 °C in 5% CO_2_ incubator. Following 42-h maturation, cumulus cells were removed with 0.1% hyaluronidase, and oocytes with first polar body extrusion were defined as matured oocytes at the metaphase II stage.

### SCNT embryo construction and transfer

The porcine SCNT technique was performed on an inverted microscope (Nikon, Eclipse Ti2-U) equipped with micromanipulator system (Narishige, NTX-N4) and CellTram 4r Air/Oil injection tube (Eppendorf). Briefly, matured oocytes were first enucleated by aspirating the first polar body and adjacent cytoplasm through a 20-μm glass pipette. Donor fibroblast cells were then injected into the perivitelline space of enucleated oocytes through the same pipette. Reconstructed oocytes were finally fused and activated by two direct current pulses (640 V, 40 µs) on Electro-Cell Manipulator (BTX, ECM2100) and cultured in porcine zygote medium-3 medium [16] at 38.5 °C in a 5% CO_2_ incubator.

Porcine SCNT embryos were transferred to the oviducts of surrogate sows at 20–24 h post-activation. Pregnancy was diagnosed by ultrasonography at 28–30 d after embryo transfer. All cloned piglets were delivered either by natural birth or cesarean section on d 114 of gestation and were subsequently examined for the growth performance after weaning.

### Growth performance evaluation

Growth performance was assessed according to the GGP Breeding Pig Performance Testing Protocol issued by the COFCO Breeding Department. Growth traits included age at 100 kg (AGE), backfat thickness (BF), loin muscle area (LMA), and feed conversion ratio (FCR). AGE: replacement pigs weighing between 90 and 110 kg were weighed using an electronic scale after a 24-h fast. The measured body weight was adjusted to the age at 100 kg body weight using breeding evaluation software. BF and LMA: backfat thickness and loin muscle area were measured simultaneously with the 100 kg body weight assessment using a B-mode ultrasound scanner. Measurements were taken at the left side between the third and fourth last ribs, approximately 5 cm from the dorsal midline. FCR: feed conversion ratio was calculated as the total feed intake divided by the total body weight gain during the growth period. Feed intake and body weight gain of boars from 40 to 100 kg were automatically recorded using an electronic feeding station.

### Embryo biopsy manipulation

Embryo biopsy was performed on the same micromanipulator system as the SCNT technique, equipped with a dynamic laser targeting system (Hamilton Thorne, XYRCOS). Embryos cultured to the 8-cell (d 3), 16-cell (d 4) and blastocyst (d 6.5) stages were placed into a drop of manipulation medium [16] in a 60-mm Petri dish. For 8-cell and 16-cell embryos, the zona pellucida was thinned by laser, and a single blastomere was aspirated using a 30-μm glass pipette. For blastocyst biopsy to obtain TE cells, the zona pellucida of day-5.5 early blastocysts were drilled by laser, followed by a further 24-h culturing to allow TE cell hatching. These hatched TE cells were then aspirated by the glass pipette, and separated by laser cutting. All biopsied cells were transferred into PCR tubes containing 4 μL PBS for WGA, and the manipulated embryos were returned to culture in an incubator to assess developmental competence.

### Immunofluorescence staining

TE biopsy cells or embryos were first washed three times (5 min each) in washing buffer [0.01% (v/v) Triton X-100 in PBS]. Fixation was carried out in PBS containing 4% (w/v) paraformaldehyde at 4 °C overnight or at room temperature for 30 min, followed by ten washes in washing buffer. The nuclei of TE biopsy cells were then dyed by 4',6-diamidino-2-phenylindole (DAPI) staining solution (Beyotime, P0131), and captured using an inverted microscope equipped with fluorescence illumination system (Nikon, Eclipse Ti2-U). Embryos were permeabilized in PBS containing 1% (v/v) Triton X-100 at room temperature for 30 min, followed by ten washes in washing buffer. And then embryos were blocked in PBS containing 3% (w/v) bovine serum albumin for 2 h at room temperature. Primary antibodies (anti-CDX2, Biogenex, MU392A-UC, 1:50; KLF4, Abcam, ab215036, 1:200) were diluted in blocking buffer and incubated with embryos for 24 h at 4 °C. After ten washes, embryos were placed into diluted secondary antibodies (anti-mouse IgG 488, Abbkine, A23210, 1:500; anti-rabbit IgG 549, Abbkine, A23320, 1:500) for 1 h at room temperature, followed by another ten washes. Finally, embryos were incubated in DAPI staining solution for 3 min and washed ten times before mounting in a 2-µL drop of mounting medium (Beyotime, P0126) on a glass slide. Fluorescence images were captured using the same microscope mentioned above.

### DNA extraction and whole-genome amplification

Genomic DNAs from bulk somatic cells were extracted using the TIANamp Genomic DNA Kit (TIANGEN, DP304) following the manufacturer’s protocols. For low-input somatic cells and TE biopsy cells, WGA was performed by multiple displacement amplification (MDA) method using the REPLI-g Single Cell Kit (QIAGEN, 150343) according to the manufacturer’s standard protocols on ProFlex PCR system (Applied Biosystems). PCR products were purified using NucleoSpin Gel and PCR Clean-up Kit (MACHEREY–NAGEL, 740609.50) following the manufacturer’s protocols. The quality and concentration of the extracted genomic DNAs were quantified using NanoDrop 2000 Spectrophotometer (Thermo Scientific). The total DNA yield was calculated by multiplying the measured DNA concentration (ng/μL) by the final elution volume (50 μL) after purification. For gel electrophoresis, 60 ng of each genomic DNA sample was mixed with DNA loading buffer and loaded onto a 0.8% agarose gel, followed by electrophoresis at 170 V for 25 min, and captured by BioDoc-It Imaging System (UVP).

### SNP chip-based genotyping

Genotyping was performed using a commercial Pig 80 K Functional Variants Genotyping Array (Wuhan Yingzi Gene Technology Co., Ltd.), which targets 80,483 functional SNPs. Raw genotyping data were processed and annotated based on the *Sus scrofa* reference genome assembly Sscrofa11.1, with functional annotation conducted using snpEff (v5.1). The SNP call rate was calculated as the proportion of successfully called SNP loci relative to the total number of SNPs included in the 80 K array. Genotype concordance was assessed by pairwise comparison of SNP genotypes between the embryo-derived data with different MDA conditions and the control group with the standard MDA condition in Fig. 2 and Fig. S1, or between three types of cell samples (including bulk cells, low-input cells, and TE biopsy cells) and their corresponding donor boars in Fig. 3. The concordance rate was defined as the ratio of concordant SNP calls to the total number of SNP loci for each comparison group.

### Estimation of genetic parameters

To assess the reliability of EGS, genomic analyses were performed using HiBLUP (High-performance integrated BLUP) software (v1.3.2) [17] and HiBayes package (v3.0.1) [18]. Raw SNP genotyping data were subjected to quality control prior to downstream analyses. SNPs with a call rate < 95% or a minor allele frequency < 1% were excluded. The remaining high-quality SNP markers were used to calculate GEBVs for samples derived from three types of cell samples (including bulk cells, low-input cells, and TE biopsy cells) and their corresponding donor boars.

GEBVs for four key traits—AGE, BF, LMA and FCR—were estimated using two genomic prediction models: the genomic best linear unbiased prediction (GBLUP) model implemented in HiBLUP and the BayesCpi model implemented in HiBayes. The reference populations consisted of two breeds, Duroc and Yorkshire. For the Duroc population, the reference population size was 5,622 for AGE and BF, and 4,261 for FCR. For the Yorkshire population, the corresponding reference population size was 17,020 for AGE and BF, and 13,751 for FCR. In addition, GEBVs for LMA in Duroc pigs were estimated using the GBLUP model, and the reference population size was 3,497.

The GBLUP model was defined as:

$${\boldsymbol{y}}={\boldsymbol{X}}{\boldsymbol{b}}+{\boldsymbol{Z}}{\boldsymbol{\alpha}}+{\boldsymbol{e}}$$

where ***y*** is the vector of phenotypic observations; ${\boldsymbol{b}}$ is the vector of fixed effects; ${\boldsymbol{\alpha}}$ is the vector of additive genetic effects, following the multivariate normal distribution ${\boldsymbol{N}}(0,{\boldsymbol{G}}{{\boldsymbol{\sigma}}}_{{\boldsymbol{\alpha}}}^{2})$, where ${\boldsymbol{G}}$ is the genomic relationship matrix derived from genotype data, and ${{\boldsymbol{\sigma}}}_{{\boldsymbol{\alpha}}}^{2}$ is the additive genetic variance; ${\boldsymbol{e}}$ is the vector of random residuals distributed on an independent and identical normality of ${\boldsymbol{N}}(0,{\boldsymbol{I}}{{\boldsymbol{\sigma}}}_{{\boldsymbol{e}}}^{2})$; ***X*** and ***Z*** are the incidence matrices relating observations to fixed and additive genetic effects, respectively.

The BayesCpi model was defined as:

$${\boldsymbol{y}}={\boldsymbol{X}}{\boldsymbol{b}}+{\boldsymbol{M}}{\boldsymbol{\beta}}+{\boldsymbol{e}}$$

where ***y*** is the vector of phenotypic observations; ***X*** is the design matrix for fixed effects with corresponding coefficients ***b***; ***M*** is the additive coding genotype matrix (i.e., 0, 1, 2 for the genotype AA, AB, BB); the marker effect ${\boldsymbol{\beta}}$ is assumed to follow a point-normal mixture prior:

$${{\boldsymbol{\beta}}}_{{\boldsymbol{j}}}\sim{\boldsymbol{\pi}}{{\boldsymbol{\delta}}}_{0}+\left(1-{\boldsymbol{\pi}}\right){\boldsymbol{N}}(0,{{\boldsymbol{\sigma}}}_{{\boldsymbol{\beta}}}^{2})$$

where ${{\boldsymbol{\delta}}}_{0}$ denotes a point mass at zero. Thus, marker ${\boldsymbol{j}}$ has no effect with probability ${\boldsymbol{\pi}}$, and has a non-zero effect drawn from a normal distribution with probability $1-{\boldsymbol{\pi}}$. This assumption reflects that only a subset of markers contributes to the genetic variation of a complex trait. The residual term ***e*** is assumed to follow a normal distribution ${\boldsymbol{N}}(0,{{\boldsymbol{\sigma}}}_{{\boldsymbol{e}}}^{2})$.

The heritability was defined as:

$${{\boldsymbol{h}}}^{2}=\frac{{{\boldsymbol{\sigma}}}_{{\boldsymbol{\alpha}}}^{2}}{{{\boldsymbol{\sigma}}}_{{\boldsymbol{\alpha}}}^{2}+{{\boldsymbol{\sigma}}}_{{\boldsymbol{e}}}^{2}}$$

where ***h***^***2***^ is the narrow-sense heritability, and all the above genetic parameters were estimated using HiBLUP software.

For each trait and each comparison group, Pearson correlation coefficients between GEBVs derived from three types of cell samples and those from the corresponding donor boars were calculated in R-based on one single prediction model.

### Statistics analysis

Statistical results were presented as mean ± standard deviation (SD) and visualized using bar charts. Statistical analyses were performed using GraphPad Prism (version 9.0.0). For comparisons between two groups, statistical significance was assessed using two-sided Student’s *t*-tests. An unpaired Student’s *t*-test was applied for comparisons between independent groups. A paired Student’s *t*-test was applied for paired experimental designs, where measurements were obtained from the same individuals and the matched samples. For comparisons among three or more groups, statistical significance was assessed using one-way analysis of variance (ANOVA) with Dunnett’s or Tukey’s multiple comparison test. *P* < 0.05 indicates a significant difference, while *P* > 0.05 indicates no significant difference.

## Results

### Application of cell biopsy in porcine SCNT embryo

**Fig. 1:**
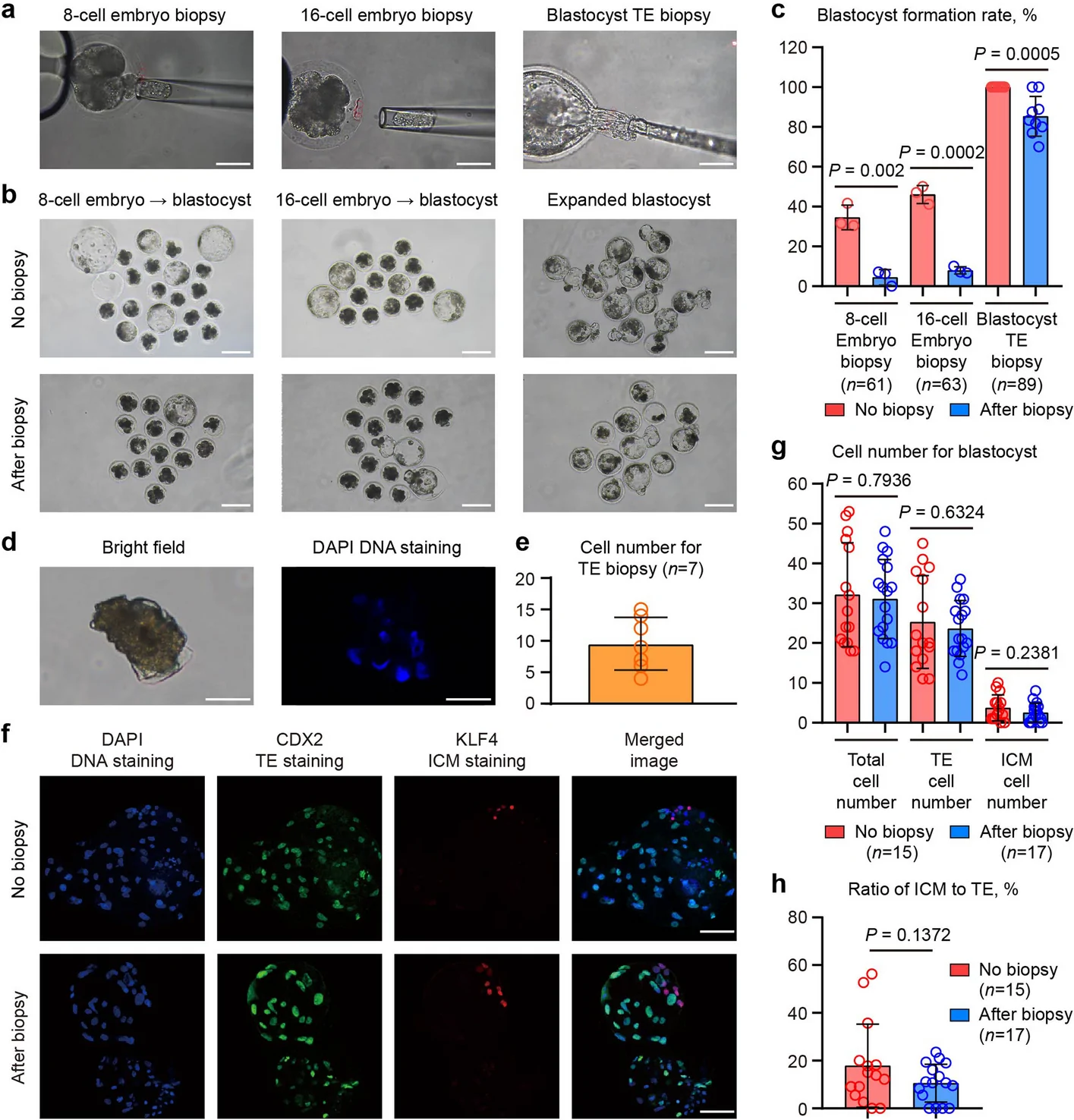
Establishment of porcine SCNT embryo biopsy manipulation. **a** Bright field images for biopsy manipulation on porcine SCNT embryos at the 8-cell, 16-cell and blastocyst stages. Scale bars, 50 μm. **b** Bright field images for blastocyst formation after biopsy manipulation on porcine SCNT embryos at the 8-cell, 16-cell, and blastocyst stages. Scale bars, 200 μm. **c** Bar plot showing the blastocyst formation rates after biopsy manipulation on porcine SCNT embryos at the 8-cell (*n* = 61), 16-cell (*n* = 63), and blastocyst (*n* = 89) stages. **d** Bright field and DNA staining images of TE biopsy cells derived from porcine SCNT blastocysts. Scale bars, 20 μm. **e** Bar plot showing the cell number of TE biopsy cells (*n* = 7) derived from porcine SCNT blastocysts. **f** Immunofluorescence staining of CDX2 (green), KLF4 (red) and DAPI (blue) in porcine SCNT blastocysts with or without biopsy manipulation. Scale bars, 50 μm. **g** Bar plot showing the total cell number (blue), TE cell number (green), ICM cell number (red) of each porcine SCNT blastocyst with (*n* = 17) or without (*n* = 15) biopsy manipulation. **h** Bar plot showing the ratio of ICM cells to TE cells in porcine SCNT blastocyst with (*n* = 17) or without (*n* = 15) biopsy manipulation. Data are presented as mean ± SD. *P* values were calculated using two-sided unpaired Student’s *t*-test

We first performed cleavage embryo biopsy to assess the influence on porcine preimplantation development. After laser-assisted drilling on the zona pellucida, a single blastomere was aspirated from embryos at the 8-cell and 16-cell stages (Fig. 1a). Following biopsy, embryos were returned to the incubator to assess their developmental potential to the blastocyst stage. The results (Fig. 1b and c) demonstrated a significant decrease in the blastocyst formation rates of biopsied embryos at both stages (8-cell biopsy: 4.46% ± 3.89%; 16-cell biopsy: 7.93% ± 1.80%) compared to the non-biopsied controls (8-cell control: 34.56% ± 6.18%; 16-cell control: 46.08% ± 4.49%). These findings show that the removal of a single blastomere from porcine 8-cell and 16-cell embryos impairs their subsequent preimplantation development. It suggests that the cleavage stage of porcine early embryos is highly sensitive to biopsy manipulation.

We then performed biopsy on porcine day-6.5 blastocysts to examine if it could obtain viable embryos after such manipulation (Fig. 1a). First, the zona pellucida of day-5.5 early blastocysts was drilled by laser to help hatching for TE cells. Second, the hatched TE cells of day-6.5 blastocysts were aspirated by the biopsy pipette, and then cut by laser. Third, the remaining blastocysts were cultured to evaluate their developmental competence after biopsy. After 24-h culturing, the re-expanded rate of blastocysts was 83.79% ± 8.38% (Fig. 1b and c). Therefore, laser-assisted TE biopsy exhibits less developmental impact than blastomere biopsy for porcine early embryos.

To quantify the sampling yield and assess its impact on embryo integrity, a comprehensive quality analysis was conducted. Direct labelling of nuclear DNA with DAPI confirmed that an average of approximately 9.5 TE cells were obtained by TE biopsy (Fig. 1d and e), meeting the cell demand (5–10 TE cells) for preimplantation genetic testing (PGT) in human blastocysts [19]. Subsequently, immunofluorescence staining was employed for comparison of cell composition between the biopsied and non-biopsied control blastocysts. Key parameters quantified include the total cell number (DAPI-positive), TE cell number (CDX2-positive), inner cell mass (ICM) cell number (KLF4-positive), and the ICM/TE ratio [20, 21]. Statistical analysis revealed no significant differences (*P* > 0.05) between the biopsied and non-biopsied control groups for any of these quality parameters (Fig. 1f and g). Collectively, we determine that TE biopsy from porcine blastocysts is a robust and compatible cell sampling technique for EGS.

### Optimization of the multiple displacement amplification assay

**Fig. 2:**
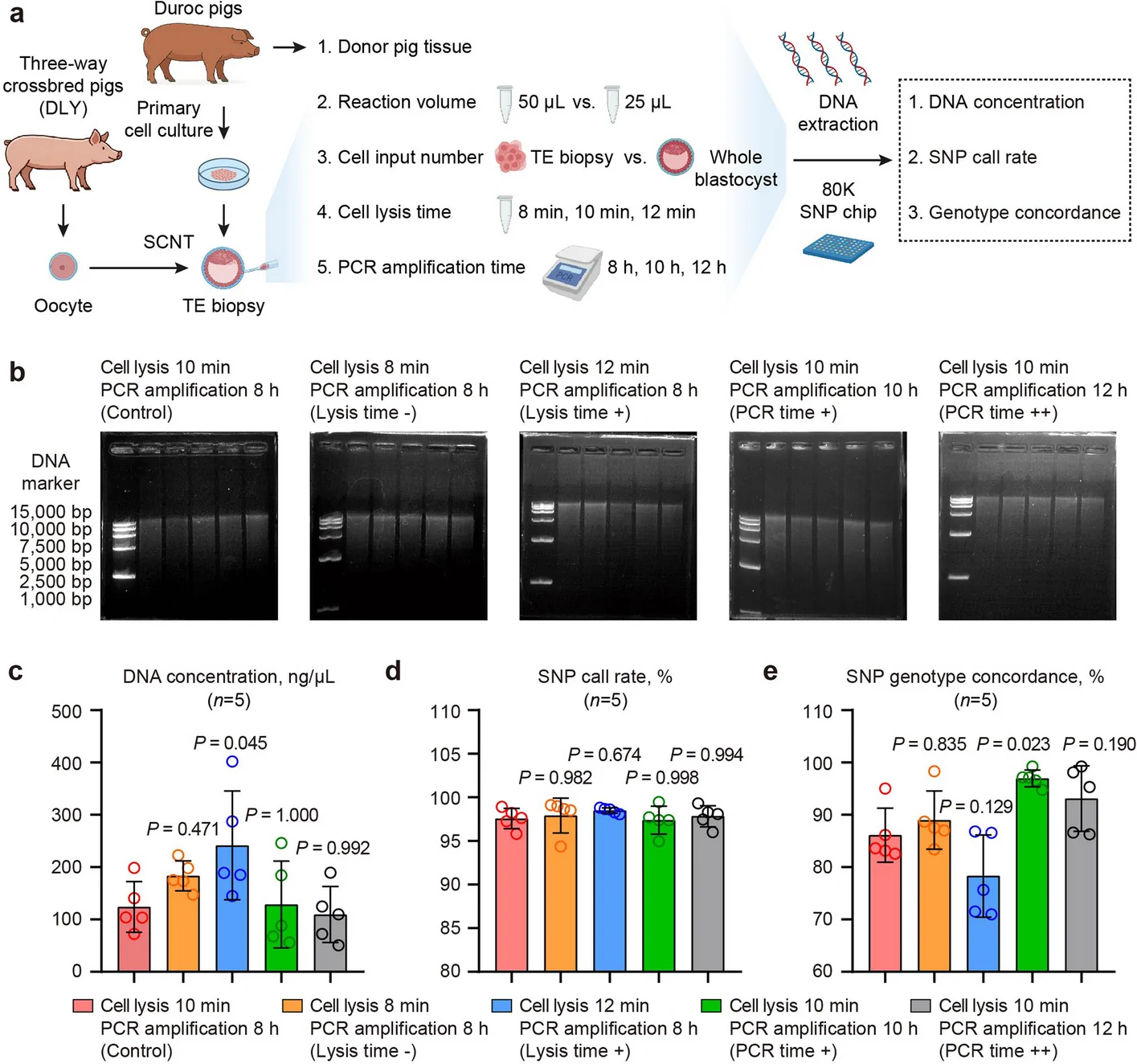
Optimization of the genome amplification assay for porcine SCNT embryos. **a** Schematic workflow of SCNT embryo sampling, MDA optimization and chip detection for porcine TE biopsy cells under different MDA conditions. MDA conditions of the control group are 25-μL reaction volume, TE biopsy cells, 10-min cell lysis and 8-h PCR amplification. **b** Agarose gel electrophoresis of embryonic genomes amplified under different MDA conditions. The first lane in each image is the DNA marker, and the other lanes in each image are embryonic genomes derived from five donor pigs as replicates. **c** Bar plot showing the DNA concentration of embryonic genomes (*n* = 5) amplified under different MDA conditions. **d** Bar plot showing the SNP call rate of embryonic genomes (*n* = 5) amplified under different MDA conditions. **e** Bar plot showing the SNP genotype concordance of embryonic genomes (*n* = 5) amplified under different MDA conditions. Data are presented as mean ± SD. *P* values were calculated using one-way ANOVA with Dunnett’s multiple comparison test when compared to the control group

Previous experiments indicated that MDA-based WGA of low-input cell samples (15 cells) achieved steady DNA output with the highest call rates and the lowest error rates of SNP genotyping [22]. We thus performed MDA to amplify genomic DNAs derived from porcine TE biopsy cells: 1) five fibroblast cell strains, with known SNP chip data from five individual candidate pigs, were chosen to construct SCNT embryos; 2) TE biopsy cells, collected from SCNT blastocysts, were conducted MDA assay and 80 K chip-based genotyping; and 3) genotype concordance was checked by comparing the chip data from the amplified embryonic DNAs with the chip data derived from the candidate pigs (Fig. 2a). Additionally, we systematically optimized the MDA protocol to enhance the yield and genotyping accuracy of porcine embryonic DNA products.

First, we investigated the effects of amplification volume on DNA production and SNP detection. Gel electrophoresis (Fig. 2b and Fig. S1a, S5) showed evident DNA bands around 15,000 bp, with smeared bands from about 2,500 to 15,000 bp, as previously reported [23]. The PCR concentration of 50-μL system was 163.16 ± 69.04 ng/μL, higher than the 25-μL system (123.76 ± 48.34 ng/μL), but the difference was not statistically significant (*P* > 0.05) (Fig. S1b). The call rates of 50-μL and 25-μL systems were 97.46% ± 1.10% and 97.57% ± 1.16%, respectively (Fig. S1c), and the genotype concordances were 82.94% ± 2.12% and 86.09% ± 5.16%, respectively (Fig. S1d). It suggests that the reaction system can be cut in half, from 50 to 25 µL, without compromising the DNA yield and the genotyping results. Next, we performed MDA assay and chip-based genotyping using the whole SCNT blastocyst (average 40 cells), and compared the results with those obtained from TE biopsy cells (average 9.5 cells). The results showed no significant differences in DNA concentration and genotyping parameters (Fig. S1e–g), indicating that TE biopsy cells are sufficient for SNP detection and analysis.

We then adjusted the timing for cell lysis (8 min, 10 min, 12 min) and PCR amplification (8 h, 10 h, 12 h) in MDA assay. The control condition was defined as a 25-μL reaction system with 10-min cell lysis followed by 8-h amplification based on the standard MDA protocol. Four experimental groups were established to test the timing for cell lysis and PCR amplification, in which all other reaction conditions remained identical to the control group. Gel electrophoresis confirmed that all optimization conditions yielded high-molecular-weight DNAs (2,500 to 15,000 bp) (Fig. 2b and Fig. S5). Here, we found that extending the cell lysis time to 12 min significantly increased DNA concentration (241.60 ± 103.85 ng/μL) compared to the control (*P* < 0.05) (Fig. 2c). In contrast, extending amplification time from 8 to 10 h and 12 h resulted in an unchanged DNA concentration (Fig. 2c). Despite these, genotyping call rates remained consistently high (> 95%) across all groups with no significant differences (*P* > 0.05; Fig. 2d). However, genotype concordance showed a distinct pattern (Fig. 2e). The concordances of the 8-min, 10-min, and 12-min lysis groups were 88.99% ± 5.57%, 86.09% ± 5.16%, and 78.30% ± 7.61%, respectively, with no significant differences (*P* > 0.05). In addition, the 10-h amplification group achieved a genotype concordance of 96.96% ± 1.59%, a substantial increase over the 8-h control (*P* < 0.05), while a 12-h extension yielded a similar concordance (93.11% ± 6.25%) to the 8-h control (*P* > 0.05). In sum, we determine that 10-min cell lysis, 10-h PCR amplification and a 25-μL reaction system, are the optimized MDA conditions for porcine embryo genotyping.

### Evaluation of DNA yields from different cell types

**Fig. 3:**
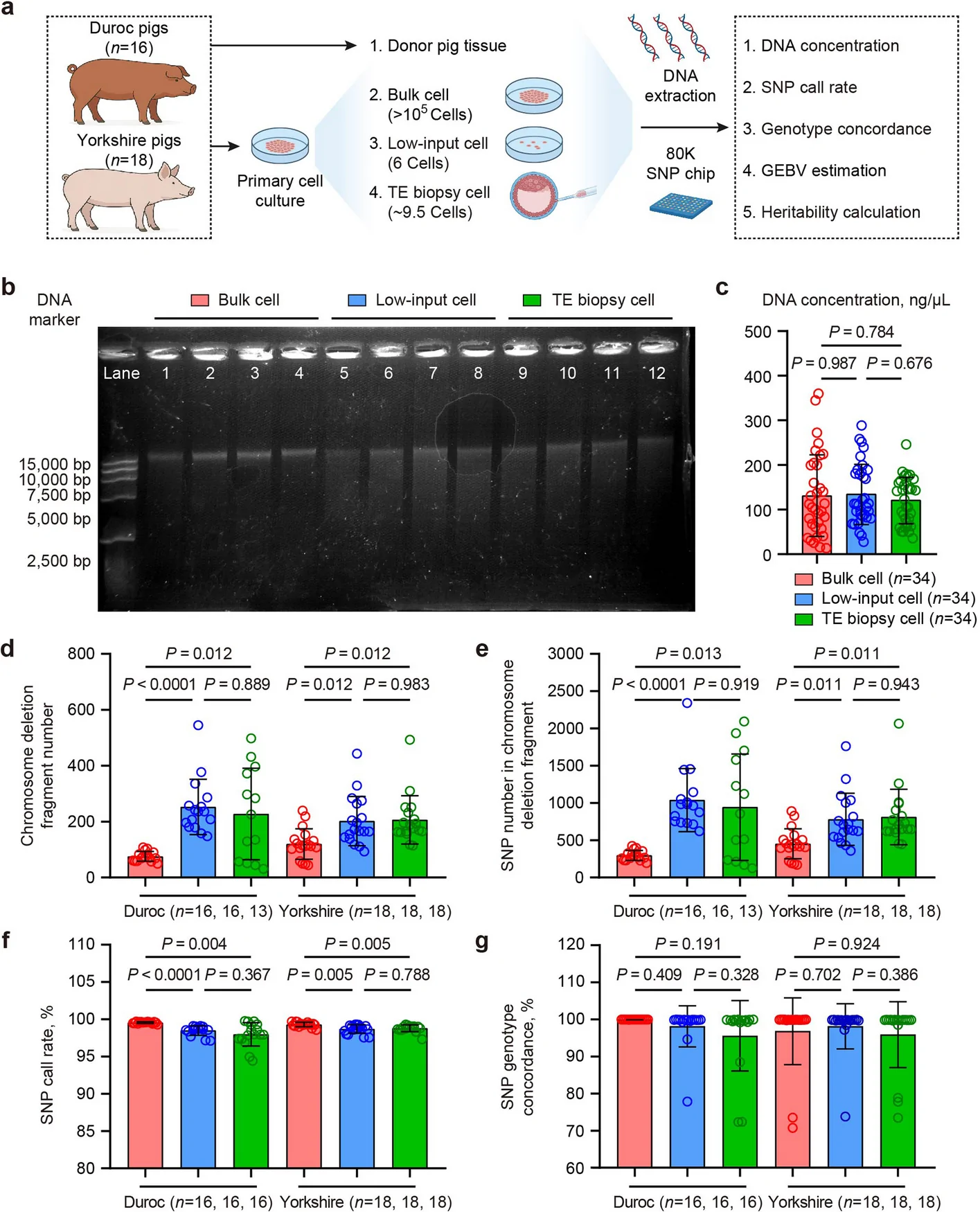
Evaluation of porcine embryo genomic selection based on MDA amplification and SNP array genotyping. **a** Schematic workflow of chip detection using four types of DNAs derived from tissue, bulk cells, low-input cells and TE biopsy cells of Duroc (*n* = 16) and Yorkshire (*n* = 18) pigs. **b** Agarose gel electrophoresis of DNA products derived from bulk cell (Lane 1–4), low-input cell (Lane 5–8) and TE biopsy cell (Lane 9–12) groups. The DNA marker is shown on the left. **c** Bar plot showing the DNA concentration among the bulk cell (*n* = 34), low-input cell (*n* = 34) and TE biopsy cell (*n* = 34) groups. **d** Bar plot showing the chromosome deletion fragment number among the bulk cell, low-input cell and TE biopsy cell groups of Duroc and Yorkshire pigs. Three outliers of TE biopsy cell group in Duroc pigs are deleted. **e** Bar plot showing the SNP number in chromosome deletion fragment among the bulk cell, low-input cell and TE biopsy cell groups of Duroc and Yorkshire pigs. Three outliers of TE biopsy cell group in Duroc pigs are deleted. **f** Bar plot showing the SNP call rate among the bulk cell, low-input cell and TE biopsy cell groups of Duroc and Yorkshire pigs. **g** Bar plot showing the SNP genotype concordance among the bulk cell, low-input cell and TE biopsy cell groups of Duroc and Yorkshire pigs. Data are presented as mean ± SD. *P* values were calculated using one-way ANOVA followed by Tukey’s multiple comparison test

To systematically assess the genotyping difference between porcine embryos and their progenies, we still used the SCNT technique to obtain TE biopsy cells from cloned blastocysts, which were derived from somatic cells collected from 16 Duroc pigs and 18 Yorkshire pigs with known 80 K chip data (Fig. 3a). Primary cells were then divided into three groups: 1) bulk cell group (> 10^5^ somatic cells); 2) low-input cell group (6 somatic cells, within the PGT validated cell number range [24]); and 3) TE biopsy cell group (average 9.5 embryonic cells). The genomic DNAs from the low-input and TE biopsy cell groups were amplified by modified MDA assay mentioned in Fig. 2, while the genomic DNAs from the bulk cell group were directly extracted by the kit (Fig. 3a).

We thus examined the DNA yields from these three groups. Gel electrophoresis results demonstrated that high-quality genomic DNAs were obtained across all groups, showing the main DNA bands around 15,000 bp (Fig. 3b and Fig. S5). Interestingly, there was a single DNA band in the bulk cell group (Fig. 3b, Lane 1–4). However, DNA bands were smeared in the low-input cell (Fig. 3b, Lane 5–8) and TE biopsy cell (Fig. 3b, Lane 9–12) groups, which is the DNA feature for MDA PCR products [23]. Quantitative analysis (Fig. 3c) displayed comparable DNA concentrations among the bulk cell (131.28 ± 91.60 ng/μL), low-input cell (134.08 ± 67.54 ng/μL) and TE biopsy cell (120.22 ± 52.08 ng/μL) groups. Notably, although the total DNA yield from the TE biopsy cell group (6.01 ± 2.60 μg) was slightly lower than that of the bulk cell (6.56 ± 4.58 μg) and low-input cell (6.70 ± 3.38 μg) groups, the insignificant difference (*P* > 0.05) among these three groups demonstrated the feasibility of MDA method to obtain sufficient genomic DNAs from porcine early embryos.

### Evaluation of SNP genotyping performance from different cell types

We next generated genotype data by using high-density 80 K SNP array for both Duroc and Yorkshire pigs across the bulk cell, low-input cell, and TE biopsy cell groups (Fig. 3a). First, genomic integrity of chip data in each group was examined (Fig. 3d and e). Chromosome loss regions were defined when at least three consecutive informative SNPs were missing, as previously reported [25]. Thus, we found 227.08 ± 164.16 segments (including 943.85 ± 712.85 SNPs) and 206.00 ± 86.40 segments (including 811.50 ± 372.31 SNPs) were lost in Duroc and Yorkshire TE biopsy cell groups, respectively (Fig. 3d and e). The number of chromosome deletion fragments in the low-input and TE biopsy cell groups was significantly higher (*P* < 0.05) than in the bulk cell group, suggesting a limitation of amplified integrity of the MDA method. Additionally, while the bulk cell group exhibited significantly higher (*P* < 0.05) genotyping call rates than the low-input and TE biopsy cell groups, the average call rates of all groups remained above 97% (Fig. 3f). This indicates that consistently high genotyping quality was maintained across all sample types. Furthermore, genotype concordance rates were calculated between each group and their corresponding donor pigs (Fig. 3g). Within Duroc pigs, no significant differences in genotype concordances were observed among the bulk cell (99.95% ± 0.02%), low-input cell (98.13% ± 5.56%), and TE biopsy cell (95.59% ± 9.49%) groups. Meanwhile, genotype concordances also remained comparable among the three cell-type groups in Yorkshire pigs (bulk cell: 96.82% ± 8.99%; low-input cell: 98.14% ± 6.11%; TE biopsy cell: 95.90% ± 8.89%). Moreover, these concordances were also comparable between Duroc and Yorkshire pigs within each of the three cell-type groups (Fig. S2a). These results indicate that MDA-amplified DNAs, regardless of the cell type and pig breeds, support robust and reliable genotyping data, similar to the tissue DNAs derived from individual donor pigs.

### Evaluation of genomic estimated breeding values from different cell types

**Fig. 4:**
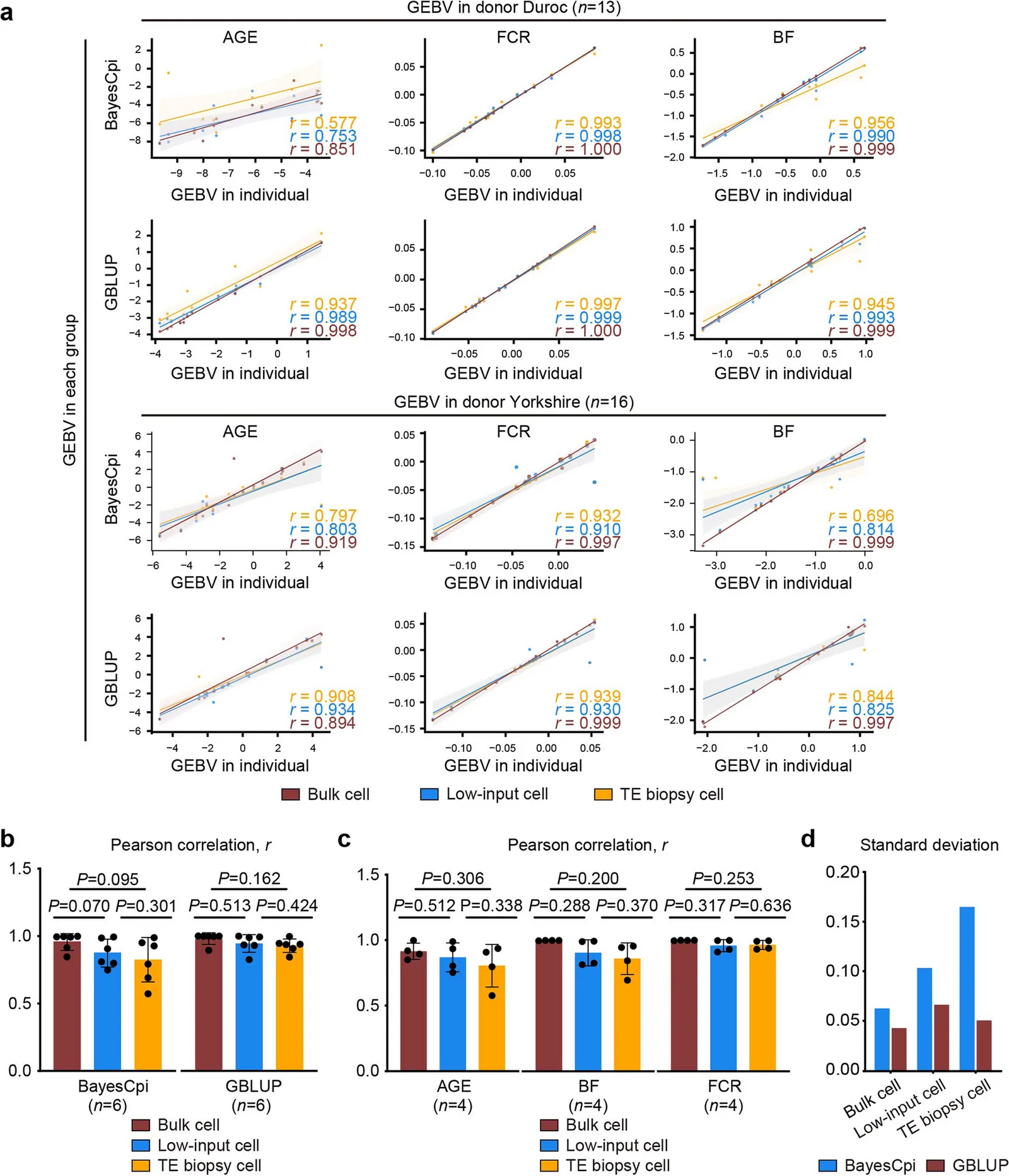
Correlation of GEBVs between SCNT embryos and the corresponding donor pigs. **a** Linear regression line plots showing the GEBVs correlation estimated by GBLUP and Bayesian models for AGE, FCR, BF traits in Duroc (*n* = 13) and Yorkshire (*n* = 16) pigs among the bulk cell, low-input cell, and TE biopsy cell groups. Pearson correlation coefficients (*r* values) are shown. **b** Comparison of Pearson correlation coefficients among the bulk cell, low-input cell, and TE biopsy cell groups under BayesCpi (*n* = 6) and GBLUP (*n* = 6) models. **c** Comparison of Pearson correlation coefficients among the bulk cell, low-input cell, and TE biopsy cell groups in AGE (*n* = 4), BF (*n* = 4), and FCR (*n* = 4) traits. **d** Standard deviations of Pearson correlation coefficients obtained from BayesCpi and GBLUP models across the three cell types. Data are presented as mean ± SD. *P* values were calculated using one-way ANOVA followed by Tukey’s multiple comparison test

Next, the genotyping chip data from Duroc and Yorkshire bulk cell, low-input cell and TE biopsy cell groups, with concordance rates above 90%, were used to calculate GEBVs. GEBVs for three porcine production traits, age at 100 kg (AGE), feed conversion ratio (FCR), and backfat thickness at 100 kg (BF), were calculated using both the GBLUP and Bayesian models under the reference database (Fig. 4a).

Overall, the GEBVs derived from all three cell types exhibited positive correlations with those of the corresponding donor pigs. Under the GBLUP model, the correlation coefficients for AGE, FCR, and BF in Duroc pigs ranged from 0.937–0.998, 0.997–1.000, and 0.945–0.999, respectively; the corresponding ranges in Yorkshire pigs were 0.894–0.934, 0.930–0.999, and 0.825–0.997. As for the Bayesian model, the correlation coefficients for AGE, FCR, and BF in Duroc pigs ranged from 0.577–0.851, 0.993–1.000, and 0.956–0.999, respectively, whereas those in Yorkshire pigs ranged from 0.797–0.919, 0.910–0.997, and 0.696–0.999. We then compared the GEBV correlation coefficients among the three cell types, and observed no significant differences (*P* > 0.05) between TE biopsy cells and bulk cells, regardless of the prediction models (Fig. 4b) or production traits (Fig. 4c). These comparisons indicate that genomic information obtained from amplified embryonic cells can effectively reflect the genetic breeding values of individual pigs.

In addition, we compared the GEBV correlation coefficients across breeds, prediction models, and production traits. Within any of the three cell types, no significant differences (*P* > 0.05) were observed between Duroc and Yorkshire pigs (Fig. S2b), between the GBLUP and Bayesian models (Fig. S2c), or among the three production traits (Fig. S2d). However, the GEBV correlations in certain TE biopsy groups were relatively low (e.g., Duroc AGE: 0.577; Yorkshire AGE: 0.797; Yorkshire BF: 0.696) (Fig. 4a). Notably, the standard deviations of the correlations were consistently higher for Bayesian than for GBLUP across all three cell types (Fig. 4d), indicating that GEBV correlations show greater variability under the Bayesian model. This suggests that the GBLUP model may be more stable and suitable for porcine EGS under the conditions and subjects of the current study.

The estimated genetic parameters further provide insights into the genetic architecture of each trait and facilitate the interpretation of differences in genomic prediction performance (Fig. S3). The heritability (*h*^*2*^) for BF trait was the highest in both breeds (0.535 in Duroc and 0.552 in Yorkshire). The *h*^*2*^ for the other traits were lower than the BF trait: AGE (0.276 in Duroc, 0.308 in Yorkshire); FCR (0.262 in Duroc, 0.210 in Yorkshire). The estimated genetic parameters were similar to those reported in previous studies in Duroc and Yorkshire populations [26–29]. The general similarity in genetic parameters between the two breeds indicates that the developed genetic evaluation model performed consistently in both breeds. Observed variations may be attributed to differences in population size, genetic background, and environmental factors.

### Evaluation of genomic estimated breeding values between cloned and donor pigs

**Fig. 5:**
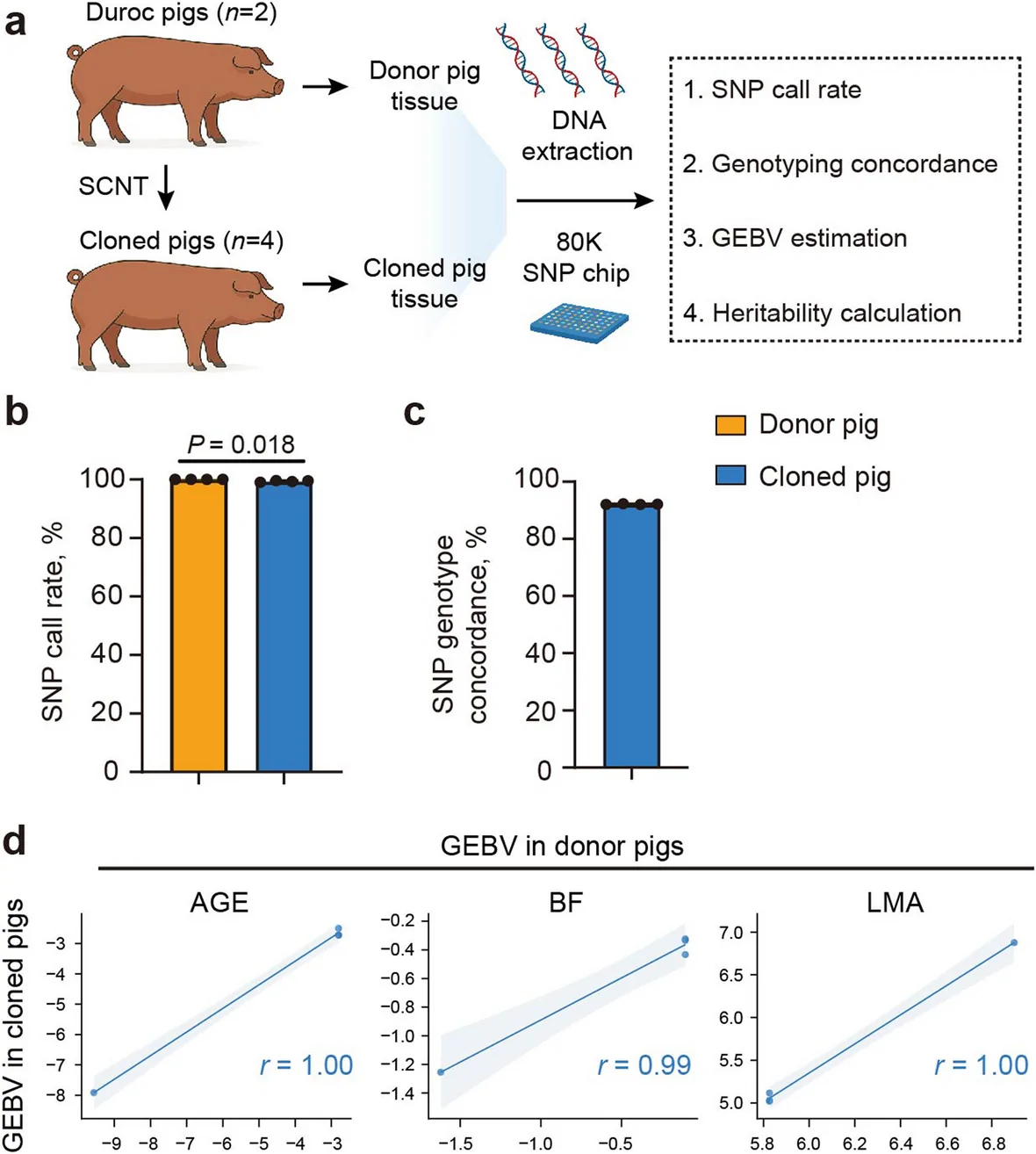
Correlation of GEBVs between cloned pigs and the corresponding donor pigs. **a** Schematic workflow of chip detection between cloned pigs (*n* = 4) and the corresponding donor pigs (*n* = 2). **b** Bar plot showing the SNP call rate between cloned pigs and the corresponding donor pigs. **c** Bar plot showing the SNP genotype concordance between cloned pigs and the corresponding donor pigs. **d** Linear regression line plots showing the GEBVs correlation estimated by GBLUP models for AGE, BF and LMA traits between cloned pigs and the corresponding donor pigs. Pearson correlation coefficients (*r* values) are shown. Data are presented as mean ± SD. *P* values were calculated using two-sided paired Student’s *t*-test

Finally, to further evaluate the feasibility of our EGS establishment by using the SCNT technique, four Duroc cloned offspring and their corresponding donor pigs were genotyped by using 80 K SNP array (Fig. 5a). The SNP chip results showed that the genotype call rates of all four cloned offspring exceeded 99%, while these rates achieved 100% for donors (Fig. 5b). The average genotype concordance between cloned and donor pigs was over 92% (Fig. 5c), and this was comparable to the genotype concordance estimated from cloned embryos (Fig. 3g).

Based on the genotype data, genetic parameters were estimated for three production traits (Fig. S4): AGE, BF and loin muscle area at 100 kg (LMA). The genetic parameters of heritability were also comparable between cloned offspring and their donors (AGE: 0.357 in clones and 0.358 in donors; BF, 0.626 in clones and 0.626 in donors; LMA, 0.543 in clones and 0.544 in donors), which is consistent with previously reported results [26, 28–30]. Next, GEBVs for AGE, BF and LMA were calculated using the GBLUP model, and a high correlation was observed between cloned and donor pigs for each trait (AGE, 1.00; BF, 0.99; LMA, 1.00) (Fig. 5d). These results indicate that cloned pigs can effectively replicate the genetic potential and growth performance from their donor pigs, and also indirectly provide evidence that establishing EGS via the SCNT technique is feasible.

## Discussion

Embryo genomic selection (EGS) is conceptually inspired by the PGT used in human assisted reproduction. Its core objective is to obtain genomic information prior to embryo transfer, which enables selective breeding at the earliest developmental stages. Unlike the PGT, which prioritizes disease avoidance, EGS in livestock breeding focuses on maximizing genetic gain and production efficiency. However, the application of EGS has long faced three questions: 1) whether embryo biopsy compromises subsequent development and viability? 2) whether embryonic genomes with low-input DNA are suitable for genotyping? and 3) whether the breeding values derived from embryos can reflect the performance of offspring? To address these questions, we established and evaluated an integrated technical workflow including embryo biopsy, embryonic genome amplification, SNP genotyping, and GEBV prediction. Our results demonstrate the high concordance of genomic information among donor pigs, the corresponding cloned embryos and their offspring, which provides the first evidence supporting the feasibility of EGS system in pig breeding.

A critical prerequisite for applying EGS is that the biopsy procedure must not compromise embryonic developmental potential. In ruminants and humans, cleavage-stage blastomere biopsy has been widely applied with minimal impact on subsequent development. For example, 78.3% of bovine embryos retained the capacity to reach the blastocyst stage after removing one blastomere from 2-cell embryos [9], and the survival rate of biopsied ovine morulae was 71.4% [31]. In humans, removal of a single blastomere at the 8-cell stage did not significantly affect birth rates compared to the control [32]. However, this approach is less suitable for porcine embryos, as cleavage-stage biopsy significantly impaired their developmental potential (Fig. 1a–c). One possible explanation is that the timing of embryonic compaction differs among species. While compaction in ruminants begins relatively late, at the 16- to 32-cell stage (d 4–5), it occurs much earlier in pigs, initiating at the 8-cell stage (d 3) [33]. Consequently, cell–cell contacts among porcine blastomeres are already established when performing blastomere biopsy, and removal of a single blastomere is more likely to disrupt the overall structural integrity of porcine embryos. Here, we proposed TE biopsy at the blastocyst stage as a more suitable sampling strategy for porcine EGS, as it provides sufficient DNAs for WGA while minimizing disturbance to developmental potential. Previous studies have demonstrated equivalent implantation rates between TE-biopsied and non-biopsied embryos in humans and cattle [34, 35], however, the long-term developmental outcomes following TE biopsy in pigs warrant further investigation.

Embryonic cells require WGA to obtain sufficient DNAs for genetic evaluation. Currently, two common WGA methods for low-input samples are MDA and multiple annealing and looping-based amplification cycles (MALBAC). MALBAC employs a looping design during pre-amplification, achieving approximately 93% genome coverage even at the single-cell level. This approach enables the detection of copy number variation (CNV) and single nucleotide variation (SNV), offering improved genome coverage compared to MDA [36, 37]. However, MALBAC inevitably introduces amplification bias during the PCR step. In contrast, MDA relies on Phi29 DNA polymerase for strand displacement synthesis at a constant temperature. It exhibits an extremely low base error rate and high amplification fidelity, making it suitable for allele genotyping and SNV identification [23]. Comparative studies have shown that, for single cells or embryonic cells, MDA consistently yields higher DNA output, higher genotype call rates, and lower mismatch rates than MALBAC [8, 9, 13, 22]. Based on these findings, we chose and modified the MDA assay for porcine embryonic genome amplification (Fig. 2). Using SNP arrays, the amplified DNAs achieved a SNP genotype call rate exceeding 97%, with high concordance in both SNP profiles and breeding values between cloned embryos and their donors. These results confirm the high integrity and reliability of embryonic DNAs amplified via the MDA method.

In this study, the Bayesian model exhibited greater fluctuation in correlations when applied to low-input WGA data (e.g., Duroc AGE: 0.577; Yorkshire AGE: 0.797; Yorkshire BF: 0.696) (Fig. 4a). This variability is likely due to the model’s sensitivity to amplification bias and missing/mismatching genotypes. BayesCpi is a Bayesian variable-selection method based on a sparse genetic architecture assumption, in which a proportion of markers is assumed to have zero effects while a subset of markers is allowed to contribute non-zero effects [38]. Therefore, its prediction performance is more sensitive to amplification-induced genotype missing or locus dropout events, because missing or erroneous genotypes at informative loci may affect the estimation of posterior marker effects and increase uncertainty in genomic prediction [39]. In contrast, GBLUP assumes an infinitesimal genetic architecture and incorporates genome-wide marker information simultaneously through the construction of a genomic relationship matrix. By integrating signals from a large number of markers rather than relying on specific loci, GBLUP partially compensates for random genotype missing and is therefore generally more robust to stochastic genotyping errors and missing data [40, 41]. Consistent with this, GBLUP showed more stable correlation coefficients with lower standard deviations across cell types, suggesting greater consistency when applied to amplified DNAs (Fig. 4). Despite these findings, future work is needed to establish strategies for amplifying low-input DNAs with higher integrity, and to construct optimized SNP chips that minimize genotyping loss from amplified DNAs.

A limitation of the present study is that the EGS workflow was established and validated using SCNT embryos rather than fertilized embryos. SCNT embryos undergo nuclear reprogramming and exhibit distinct developmental characteristics compared to fertilized embryos, such as higher rates of early embryo loss, placenta abnormalities, and lower birth rates [42, 43]. However, our results indicate that these abnormalities do not substantially influence the accuracy of SNP genotyping or GEBV estimation, as the donor somatic cells and their resulting SCNT embryos share an identical nuclear genome. Future studies will extend this workflow to fertilized embryos to evaluate its applicability in commercial breeding. The proposed procedure involves four steps: 1) flushing naturally fertilized embryos from the uterus, or performing IVF, and culturing fertilized embryos to the blastocyst stage; 2) performing EGS, including TE biopsy, MDA amplification, SNP genotyping, and GEBV prediction; 3) transferring biopsied embryos with top GEBVs to recipient sows; and 4) collecting genotype and phenotype data from the resulting piglets to analyze concordance between embryos and their offspring. This EGS workflow involves additional steps that increase technical complexity and costs compared to conventional GS, and requires technicians to perform embryo collection, micromanipulation, and transfer. However, ongoing advances in reproductive biotechnology, coupled with decreasing costs of amplification and genotyping, are expected to enhance the commercial feasibility of EGS. Furthermore, the current requirement to genotype offspring serves as a validation step for fertilized embryos. Once the improvement in average productive performance is conclusively demonstrated, the EGS workflow will require only a single genotyping at the preimplantation stage, thereby reducing the costs in pig breeding.

## Conclusions

In summary, this study established a complete workflow for porcine EGS, including genome sampling, amplification, and genetic analysis. Our results demonstrate that porcine embryonic genome can accurately predict the future production performance of individual pigs. Besides SNP array analysis, introducing whole-genome sequencing may further improve EGS accuracy. For example, combining the high-accuracy variant detection of next-generation sequencing with the long-read capabilities of third-generation sequencing would offer complementary benefits for quantitative trait locus mapping and causal variant analysis, thereby enhancing the resolution of genetic analyses for complex traits. Moreover, current research still lacks large-scale association analyses between cloned embryos and the corresponding cloned pigs, validation of EGS in normally fertilized embryos and their offspring, and other important production traits with different genetic architectures. Future studies should focus on collecting more data and addressing the bottleneck of low efficiency that currently limits the routine commercial application of EGS.

## Supplementary Information

Additional file 1: Fig. S1. Optimization of the genome amplification assay for porcine SCNT embryos. Fig. S2. Comparison of GEBV prediction correlations across breeds, genomic prediction models, and production traits. Fig. S3. Tables showing the genetic parameter estimation for AGE, FCR, and BF traits in Duroc and Yorkshire populations by using HiBLUP. Fig. S4. Tables showing the genetic parameter estimation for AGE, LMA and BF traits in cloned and donor Duroc populations by using HiBLUP. Fig. S5. Uncropped gel images in this study.

## Data Availability

The datasets used and/or analysed during the current study are available from the corresponding author on reasonable request.

## Abbreviations

AGE: Age at 100 kg
BF: Backfat thickness at 100 kg
BLUP: Best linear unbiased prediction
CNV: Copy number variation
DAPI: 4',6-Diamidino-2-phenylindole
EGS: Embryo genomic selection
FBS: Fetal bovine serum
FCR: Feed conversion ratio
GBLUP: Genomic best linear unbiased prediction
GEBVs: Genomic estimated breeding values
GS: Genomic selection
ICM: Inner cell mass
LMA: Loin muscle area at 100 kg
MALBAC: Multiple annealing and looping-based amplification cycles
MDA: Multiple displacement amplification
PBS: Phosphate buffer saline
PGT: Preimplantation genetic testing
SCNT: Somatic cell nuclear transfer
SNP: Single nucleotide polymorphism
SNV: Single nucleotide variation
TE: Trophectoderm
WGA: Whole-genome amplification
