# Supplementary material for: Establishment and validation of porcine embryo genomic selection system through SCNT technique
Source: J Anim Sci Biotechnol. 2026 Oct 8;17:190. doi: 10.1186/s40104-026-01514-8 (PMC13647512; doi:10.1186/s40104-026-01514-8)
Supplement: Supplementary file 1 [file 40104_2026_1514_MOESM1_ESM.docx]

**Supplementary Information**

**Fig. S1** Optimization of the genome amplification assay for porcine SCNT embryos. **a** Agarose gel electrophoresis of embryonic genomes amplified under 50-μL reaction volume (left) and using the whole blastocyst (right). The first lane in each image is the DNA marker, and the other lanes in each image are embryonic genomes derived from five donor pigs as replicates. **b** Bar plot showing the DNA concentration of embryonic genomes amplified 25-μL or 50-μL reaction volume. **c** Bar plot showing the SNP call rate of embryonic genomes (*n*=5) amplified under 25-μL or 50-μL reaction volume. **d** Bar plot showing the SNP genotype concordance (*n*=5) of embryonic genomes amplified under 25-μL or 50-μL reaction volume. **e** Bar plot showing the DNA concentration of embryonic genomes (*n*=5) amplified using TE biopsy cells or the whole blastocyst. **f** Bar plot showing the SNP call rate of embryonic genomes amplified using TE biopsy cells or the whole blastocyst. **g** Bar plot showing the SNP genotype concordance (*n*=5) of embryonic genomes amplified using TE biopsy cells or the whole blastocyst. Data are presented as mean ± SD. *P* values were calculated using a two-sided unpaired Student’s *t*-test

**Fig. S2** Comparison of GEBV prediction correlations across breeds, genomic prediction models, and production traits. **a** Comparison of SNP genotype concordance rates between Duroc (DU, *n*=16) and Yorkshire (LW, *n*=18) pigs among the bulk cell, low-input cell, and TE biopsy cell groups. **b** Bar plot showing the Pearson correlation coefficients between two breeds (DU, *n*=6 vs. LW, *n*=6) among the bulk cell, low-input cell, and TE biopsy cell groups. **c** Bar plot showing the Pearson correlation coefficients between two genomic prediction models (GBLUP, *n*=6 vs. BayesCpi, *n*=6) among the bulk cell, low-input cell, and TE biopsy cell groups. **d** Bar plot showing the Pearson correlation coefficients across three production traits (AGE, *n*=4; FCR, *n*=4; BF, *n*=4) among the bulk cell, low-input cell, and TE biopsy cell groups. Data are presented as mean ± SD. *P* values were calculated using a two-sided unpaired Student’s *t*-test for comparisons between breeds, using two-sided paired Student’s *t*-test for comparisons between models, and using one-way ANOVA with Tukey's multiple comparisons test for comparisons across traits

**Fig. S3** Tables showing the genetic parameter estimation for AGE, FCR, and BF traits in Duroc (DU) and Yorkshire (LW) populations by using HiBLUP. Genetic variance (Var), variance standard error (Var_SE), heritability (*h^2^*), heritability standard error (*h^2^*_SE), and likelihood ratio test *P* values [*h^2^*_Pr (Chi-square)] are shown for each trait.

**Fig. S4** Tables showing the genetic parameter estimation for AGE, LMA and BF traits in cloned and donor Duroc (DU) populations by using HiBLUP. Genetic variance (Var), variance standard error (Var_SE), heritability (*h^2^*), heritability standard error (*h^2^*_SE), and likelihood ratio test *P* values [*h^2^*_Pr (Chi-square)] are shown for each trait

**Fig. 2b Control**

**Fig. 2b Lysis time –**

**Fig. 2b Lysis time +**

**Fig. 2b PCR time +**

**Fig. 2b PCR time ++**

**Fig. 3b**

**Fig. S1a 50-μL reaction volume**

**Fig. S1a Whole SCNT blastocyst**

**Fig. S5** Uncropped gel images in this study
